# THE RELATIONSHIP OF FRAILTY, FEAR OF FALLING, AND DEPRESSION WITH HRQOL IN HIGH-RISK OLDER ADULTS

**DOI:** 10.1093/geroni/igz038.3214

**Published:** 2019-11-08

**Authors:** Michelle A McKay, Linda Copel, Catherine Todd-Magel

**Affiliations:** Fitzpatrick College of Nursing, Villanova University, Villanova, Pennsylvania, United States

## Abstract

One in four older adults fall every year. Falls result in negative outcomes including decreased health-related quality of life (HRQoL). Frailty, fear of falling, depression, and HRQoL are not routinely screened in high-risk community-dwelling older adults. Continued study of modifiable fall risk factors is warranted due to varied reported prevalence rates, inconsistent definitions and the persistent high rate of falls resulting in poor HRQoL. The purpose of the study was to determine the relationship between frailty, fear of falling, and depression with physical and mental functioning and well-being measures of HRQoL in community-dwelling older adults 55 years of age and older. A cross-sectional correlational design and chart review were conducted. The sample consisted of 84 primarily African American (81%) nursing home eligible members of the Program for All-Inclusive Care for the Elderly (PACE) program. Data were analyzed with correlational statistics, multiple linear, and hierarchical regression models. Physical functioning and well-being measures were significantly decreased when compared to the general population. Increased frailty, fear of falling, and depression were associated with decreased physical and mental well-being. In the regression model, frailty and fear of falling were significant predictors of decreased physical functioning and well-being, and depression was a significant predictor of decreased mental functioning and well-being. This study provides clarification of the relationship between frailty, fear of falling, and depression with HRQoL in high-risk older adults. Screening for common modifiable risk factors can assist in the development of targeted interventions and treatments to improve HRQoL in high-risk older adults.

